# Projected effectiveness of lung cancer screening and concurrent smoking cessation support in the Netherlands

**DOI:** 10.1016/j.eclinm.2024.102570

**Published:** 2024-04-08

**Authors:** Koen de Nijs, Kevin ten Haaf, Carlijn van der Aalst, Harry J. de Koning

**Affiliations:** Department of Public Health, Erasmus MC - University Medical Center Rotterdam, 3015 CE Rotterdam, The Netherlands

**Keywords:** Computed tomography, Lung cancer, Screening, Smoking cessation, Early detection, NELSON

## Abstract

**Background:**

The NELSON trial demonstrated a 24% intention-to-screen reduction in lung cancer mortality from regular screening with low-dose computed tomography. Implementation efforts in Europe are ongoing, but still await country-specific and NELSON-adapted estimates of the benefits and harms of screening.

**Methods:**

We use the MISCAN-Lung microsimulation model, calibrated to individual-level outcomes from the NELSON trial, to estimate the effectiveness under 100% compliance of biennial lung cancer screening with concomitant smoking cessation support for Dutch cohorts 1942–1961. The model simulates smoking behaviour, lung cancer incidence and the effects of screening and smoking cessation on lung- and other-cause mortality.

**Findings:**

We find biennial screening with eligibility criteria equal to those of the 4-IN-THE-LUNG-RUN implementation trial to reduce lung cancer mortality by 16.9% among the eligible population, equivalent to 1076 LC deaths prevented per year in the next two decades. Eligible individuals constitute 21.5% of the cohorts studied, and stand to face 61% of the projected lung cancer mortality burden in the absence of screening. 10.3 life-years are gained per prevented LC death, for 14.9 screens per life year gained. Concomitant smoking cessation interventions may increase the expected gains in life years from screening by up to 20%.

**Interpretation:**

Policy makers should imminently consider the implementation of lung cancer screening in Europe, paired with effective smoking cessation interventions. Smoking cessation interventions on their own are not estimated to yield a gain in remaining life expectancy of the magnitude offered by even a single CT screen.

**Funding:**

10.13039/501100000780European Union10.13039/501100007601Horizon 2020 grant 848294: 4-IN-THE-LUNG-RUN.

**Research in context**

**Evidence before this study**

To identify the evidence preceding this study, we searched PubMed with the search string ((Lung Cancer{MeSH}[Title/Abstract]) AND (Screening{MeSH} [Title/Abstract])) AND (Smoking Cessation{MeSH} [Title/Abstract])], limited to English-language articles published in the past 10 years (up to March 2024). The abstracts and titles (n = 373) were screened to identify studies that evaluated the effectiveness of smoking cessation and lung cancer screening. We identify 16 studies that evaluate various smoking cessation interventions within the context of lung cancer screening, which found in-person counselling and support with pharmacotherapy to yield significant increases in cessation rates.

**Added value of this study**

We add to the existing literature by providing lung cancer screening effectiveness projections incorporating NELSON evidence. We provide scenarios of stand-alone smoking cessation interventions and complementary screening- and cessation interventions, focusing on the relative benefit of a smoking cessation intervention and CT screening for screening-eligible current smokers.

**Implications of all the available evidence**

Both lung cancer screening and smoking cessation interventions have been shown to be effective public health interventions. Our study additionally shows that smoking cessation interventions may not effectively substitute lung cancer screening to combat lung cancer death and prolong life among the screening-eligible population. Policy makers and clinicians should consider lung cancer screening and smoking cessation interventions as complementary interventions.

## Introduction

In 2022, the council of the European Union issued a recommendation in favor of screening for lung cancer (LC) by means of low-dose Computed Tomography (CT),^1^ following a favorable LC-specific mortality reduction of 24% in the Dutch–Belgian lung-cancer screening trial (NELSON), as well as favorable results from other CT screening trials worldwide.^2^^,^^3^ This has led to widespread European plans, implementation trials and pilots.^4^, ^5^ However, concerns surrounding the benefit-harms trade-off are still raised, particularly with regards to false-positive rates, the life expectancy of heavy smokers, and the comparative benefit of primary prevention.^6^, ^7^, ^8^ Estimates of these elements are therefore needed to aid policy makers. That is, the expected burden of follow-up procedures and screening-related anxiety from false-positive results, the expected life-years gained among the target population, and the extent to which smoking cessation could increase or substitute health gains from screening.

To this end, we use the MISCAN-Lung microsimulation model to estimate CT screening effectiveness for LC in the Netherlands. We include scenarios with various levels of smoking cessation support, both with and without concomitant screening. Our estimates represent the first projections from a model calibrated to individual-level outcomes from the NELSON trial, including the additional benefit of the volume-based nodule management protocol.^9^

## Methods

The MISCAN-Lung model is a microsimulation model of smoking behavior, LC natural history, and screening effectiveness that has been described previously.^10^^,^^11^ The model performs simulations of individual life histories, comparing scenarios with and without public health interventions such as lung cancer screening. The model has informed 2013 and 2021 United States Preventive Services Task Force (USPSTF) recommendations^12^^,^^13^ and recommendations for Switzerland, Australia and Ontario.^11^^,^^14^^,^^15^ The model was adapted to the Netherlands by integrating smoking behavior (initiation and cessation rates, and smoking intensity) informed by Dutch health surveys from 1989 to 2020.^16^ LC epidemiology and screening effectiveness parameters were derived from individual-level outcomes from NELSON, as well as national LC epidemiology data.^9^^,^^17^ A detailed description of the model and its assumptions is provided in the Supplementary material.

A no-screening scenario was considered, as well as scenarios with smoking cessation interventions, CT screening and CT screening with integrated smoking cessation from 2022 onwards by means of the inclusion strategy maintained by the 4-IN-THE-LUNG-RUN (4ITLR) trial^4^^,^^18^: biennial screening ages 60–79 for those with 35 pack-years and no more than 10 years of smoking cessation, or otherwise a 2.6% minimum PLCOm2012^19^ LC risk score. We simulate individual life histories for Dutch cohorts 1942–1961, who would stand to become eligible in the year 2022. The older cohorts are exposed to screening until they reach the age of 80, whilst 1961-born eligibles may be screened up to 20 years.

### Statistical analysis

Scenarios with smoking cessation interventions include various levels of smoking cessation support, with odds ratios (OR) of smoking cessation taken from the literature for four cessation support modalities: web-based support (OR 1.14), telephone counselling (OR 1.21), in-person counselling (OR 1.46) and pharmacotherapy (OR 1.53).^20^ The odds-ratios are applied to the specific background cessation rates for the cohort, sex and age of the simulated individual to obtain the instantaneous probability of cessation at the moment of the intervention. Upon simulated smoking cessation, the life-history is adjusted to account for the adjusted smoking history, including the age of LC onset and LC death, as well as the other-cause mortality age. If a preclinical LC is already present, no adjustment is made to LC onset- or mortality. We evaluate scenarios in which each cessation intervention is effective with every screening round, scenarios in which cessation interventions are only applied upon entry into screening, as well as scenarios with cessation interventions in the absence of screening. As a base-case, we evaluate model outcomes assuming perfect attendance to screening and smoking cessation interventions, representing the maximal gross effectiveness under successful implementation. A scenario with a 50% attendance rate is included as a sensitivity analysis.

### Ethics statement

No identifiable information was used; therefore, no institutional review board (IRB) approval was needed.

### Role of the funding source

Our funding source had no role in the study design, nor in data collection, analysis and interpretation, nor the writing of the manuscript. All authors had full access to the data and carry the responsibility to submit for publication.

## Results

### Effectiveness of screening

**Fig. 1:**
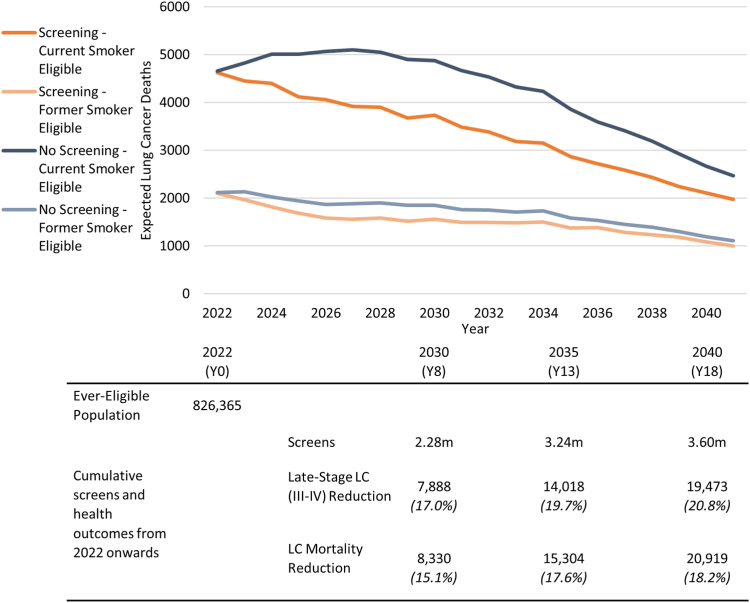
**Expected absolute number of lung cancer deaths among 1942–1961 Dutch cohorts for those eligible for screening.** Results are stratified by the smoking status upon entry into screening and shown for scenarios with and without biennial screening. Screening is applied per 4ITLR criteria,^4^^,^^18^ such that all those eligible have at least 35 pack-years or 2.6% PLCOm2012 risk,^19^ and are screened biennially from ages 60 to 79. The table below the Figure shows the cumulative number of CT screens, reduction in late stage (III-IV) lung cancers and lung cancer deaths prevented from 2022 to 2030 (after 8 years), 2035 (13 years) and 2040 (18 years), respectively.

Fig. 1 shows the expected LC deaths for the first 20 years of screening implementation, if screening were to start in 2022. In a scenario without screening, the peak of lung cancer mortality for screening-eligibles from cohorts 1942 to 1961 falls in year 2024, with 7025 total expected LC deaths. In a screening scenario, part of these LC deaths are prevented by means of early detection and treatment, such that the peak of LC mortality lies in 2022 with 6727 total deaths. By 2029, the expected annual LC deaths are reduced by 23% relative to the scenario without screening, to 5194 (1533 LC deaths averted) in this high cut-off 4ITLR strategy.

Table 1 shows the estimated effectiveness of biennial screening ages 60–79 per 4ITLR criteria, split up by subpopulation: all current smokers (including ineligibles), all former smokers (including ineligibles), all those eligible for screening, and the total population. For the total population, we project a lifetime LC incidence (from 2022 onwards) of 240 thousand cases among 3.84 million individuals born 1942–1961, associated with 218 thousand LC deaths. We find that 21.5% would be eligible for screening at some point after 2022. Assuming perfect attendance, screening is estimated to reduce LC mortality by 10.8% (23.5 thousand deaths). On average, we predict 10.3 life years gained per LC death prevented.

**Table 1:** Benefits and harms of lung cancer screening per the 4ITLR^4^^,^^18^ strategy.

∗Smoking status as determined at the simulated start of screening (2022), including never-eligible smokers.

^§^Ever-eligible for screening at the start of screening (2022), including former smokers.

^†^Biennial screening ages 60–79 for those with at least 35 packyears or 2.6% PLCOm2012 risk, and maximally 10 years of smoking cessation.

^‡^Lung Cancer.

^¶^Low-dose Computed Tomography.

^#^For the population-wide results, we also present outcomes for the scenario with pharmacotherapy smoking cessation support, applied at every screening round. For this scenario, overdiagnosis is measured as excess incidence relative to the scenario with only smoking cessation support (no screening). Results are generated for cohorts 1942–1961, with cohort sizes matched to 2021 populations by birth-year and sex for the Netherlands. Together these cohorts represent 3.85 million people as of 2022. Outcomes are tallied for the entire simulated lifetimes from 2022 onwards.

Those eligible for screening are expected to face 61% of the total LC mortality burden. Among this group, the lifetime stage distribution of LC (screen-detected and clinically detected cases combined) would shift from 23% to 41% stage I-II. The screen-detected cases are expected to be 60.5% stage I-II, with only 13.5% stage IV. This reflects that many of the cancers will still occur outside screening, given that the 1942–1961 cohorts will age out of screening within 1–20 years. The burden of screening would include 3.60 million screens over 20 years, an expense of 14.9 screens per life-year gained. Overdiagnosed cancers are estimated at 7016, one overdiagnosed LC for every 3.4 LC deaths averted. Applying false-positive rates from NELSON, we expect 40,680 false positive results, one for every 89 screening exams.

Among former smokers from cohorts 1942 to 1961, we expect 17.5% will be eligible per 4ITLR criteria. This group is projected to see a reduction of 4.6% in LC mortality from biennial screening. For current smokers, the inclusion criteria are expected to cover 74.2% of the population, with screening reducing LC mortality by 22.1%, a total of 18,974 deaths.

Our base-case analysis reports effectiveness under perfect attendance of screening. The population-level impact of screening may be reduced if eligible individuals attend their screening at a lower rate. Supplementary Table 1 reproduces the results of Table 1 when using an attendance rate of 50%. We find that the projected harms and benefits reduce slightly less than proportionally to the attendance rate, with a population-wide LC mortality reduction of 6.1%, yielding a total of 136,300 life years gained (−44.5% relative to full attendance).

### Benefits of smoking cessation

**Fig. 2:**
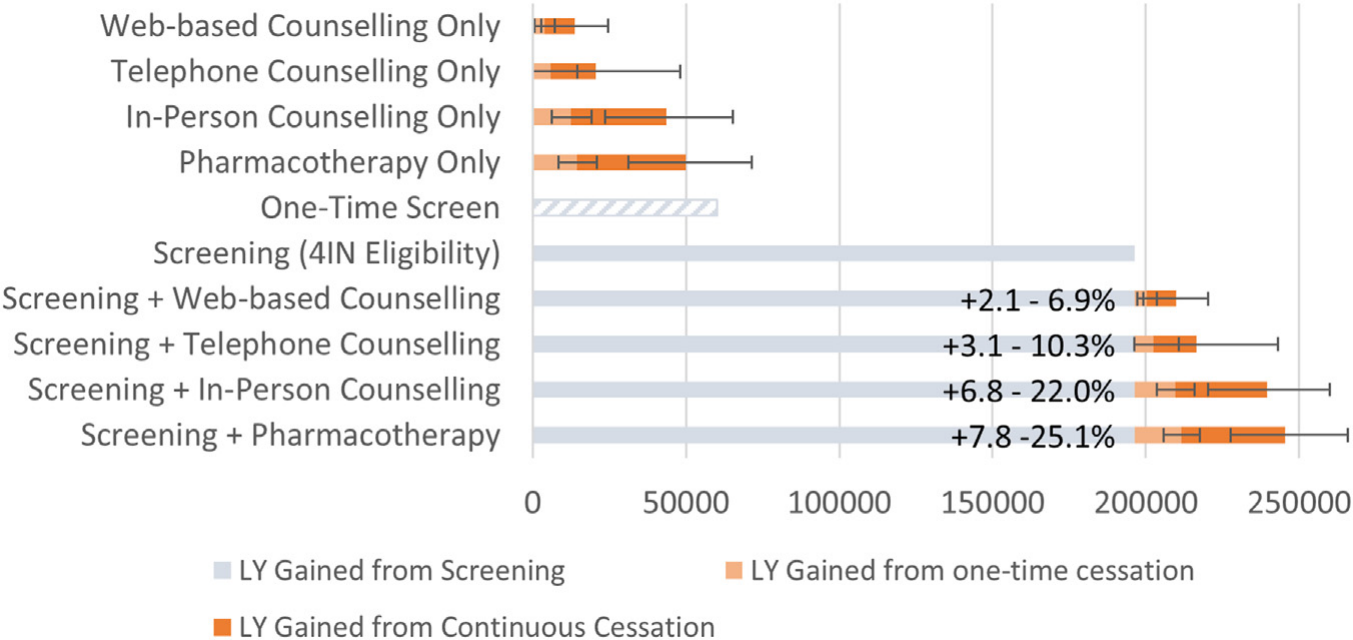
**Expected absolute number of Life-years (LY) gained among current smokers by screening intervention and supplementary or stand-alone smoking cessation support.** All strategies offer either screening or smoking cessation support to those eligible per 4ITLR∖ screening criteria^4^^,^^18^: 60–79 years old, at least 35 Packyears or 2.6% PLCOm risk.^19^ For smoking cessation, effectiveness in life years gained is shown for one-time smoking cessation support (at the start of screening), or continuous smoking cessation (with every biennial screen). Stand-alone smoking cessation support is offered under the same eligibility criteria and with the same interval (if offered continuously) as LC screening. For scenarios with both screening and smoking cessation support, the life years gained from smoking cessation relative to screening only is shown for one-time and continuous smoking cessation, respectively. For perspective, we also show the life years gained of performing a one-time screen. Confidence intervals correspond to the life years gained at the bounds of the 95% confidence interval of the Odds-Ratios of smoking cessation associated with each smoking cessation intervention.^20^

Fig. 2 shows the estimated effectiveness of screening for current smokers only, with and without smoking cessation, by measure of life-years gained relative to the scenario without any intervention. Additionally, we estimate the gains in life-years if cessation support would be offered to the 4ITLR eligible population instead of screening, maintaining the same eligibility criteria and intervention interval. We find that the largest expected gain in life-years is attributable to screening, which may be further increased up to 25.0% for continuous pharmacotherapeutic cessation support, or 7.8% if only offering this cessation intervention at the start of screening. Cessation support on its own maximally yields 67.6 thousand life years for 4ITLR eligibles, 0.20 years per eligible current smoker. We estimate 1762 lifetime LC deaths prevented for this scenario, a fraction compared to the 23,488 LC deaths prevented expected from screening (Table 1). To study the uncertainty associated with the odds-ratio applied for each smoking cessation intervention, we also evaluate life-years gained at the bounds of the 95% confidence interval reported for these modalities. These estimates are represented by the error bars in Fig. 2. We find that even in the most optimistic scenarios, smoking cessation interventions on their own do not compete with the effectiveness of screening.

## Discussion

We estimated the effectiveness of screening by means of the 4ITLR^4^^,^^18^ strategy for Dutch cohorts 1942–1961. We expect biennial screening, with 100% attendance, to reduce LC mortality by 10.8%, or 22.1% for the eligible population. This 4ITLR strategy is targeted at those with a very high LC risk, as to generate a statistically informative screening yield within a trial setting, but this eligible population accounts for 61% of the LC mortality burden. We adjust for other-cause mortality related to smoking, but still find the benefits in life-years gained substantial. About 826,000 people would apply, ultimately expecting 23,500 LC deaths prevented (gaining 10.3 years of life) and 7000 overdiagnosed cases (4.6% of all cancers among eligibles). For every 3.4 deaths averted, one overdiagnosis is expected, similar to the estimate of 3.5 for breast cancer.^21^ 19.3% of LC deaths averted are being prevented in individuals that had stopped smoking before entry. An intensive smoking cessation programme could optimally add another 20.4% to gains in life expectancy.

These estimates are consistent with previous estimates for other countries,^11^^,^^12^^,^^14^ as well as results from NELSON and NLST trials.^2^^,^^22^ Expected gains of LC screening may be larger when considering less restrictive strategies; the USPSTF recommends annual screening ages 50–80 from 20 packyears, and the UK Targeted Lung Health Check uses a risk threshold of 1.51% rather than the 2.6% employed by the 4ITLR strategy. Population screening may consider more frequent and widespread screening. However, for resource-constricted countries or policymakers wanting to start screening tentatively, biennial screening for the highest-risk group may offer a feasible initial implementation strategy. We find screening effectiveness to be sensitive to the attendance rate, reducing lifeyears gained by 44% if attendance is only 50%. Implementation efforts of screening should focus on encouraging consistent repeat attendance in order to yield the benefits promised by trial results.

Additionally, we evaluated the comparative and incremental effectiveness of smoking cessation interventions. We found that independently, smoking cessation does not offer the gains in life-expectancy estimated for a screening-only scenario. However, screening may valuably be supplemented with a pharmacotherapy intervention, which we estimate yields 7.8% extra life years gained among current smokers if offered at the start of screening. On its own, we do not find primary prevention to be a substitute for screening for the population of 4ITLR eligibles. Current smokers eligible for screening under these criteria already have a high probability of developing LC at the moment of their cessation intervention. The benefit of smoking cessation for LC prevention is therefore limited for this group, and will be much greater when applied to younger smokers with lower cumulative LC risk. This may also be because estimates of smoking cessation effectiveness from the literature^20^ result in low instantaneous cessation probabilities, consistent with other literature showing inconsistent effectiveness of screening-concomitant smoking cessation support.^8^ This underwrites the need for further research into smoking cessation effectiveness among screening populations.

Our estimates are limited by studying gross effectiveness, rather than overall cost-effectiveness, which is an evident avenue for further research. Other studies have also shown that screening effectiveness may vary by inclusion criteria and frequency and duration of screening.^23^ Exclusion of those with limited life expectancy was also not accounted for, whilst this has been shown to reduce overdiagnosis and improve effectiveness.^24^ We also do not incorporate Quality of Life in the presented analysis, which should be considered for future estimations of the optimal screening strategy.^25^ Additionally, the cohorts incorporated in our analysis are currently eligible, but gradually become ineligible within the forecasting horizon. Further estimates should also consider the future generations eligible to attend screening, current smokers born in the 1970s and 1980s. For current smokers who are not yet screening eligible, the value of screening compared to smoking cessation support thus requires further evaluation. Because we assume perfect attendance, our results should only be interpreted as the expected benefit for the individual who attends screening. Population-wide benefits will be restricted by real-world rates of screening attendance. Targeted lung health checks in the UK, as well as the 4ITLR trial, show promising results in reaching the lower socioeconomic status, commonly considered the hard-to-reach segment of potential screening eligibles.^4^^,^^18^ Finally, we are limited by offering extrapolations of trial results by means of a microsimulation model. As population-level screening is increasingly implemented in the general population, empirical data may be used to inform whether the benefits and harms of screening conform to projections based on the trial setting.

To conclude, we estimate that LC screening in a rather restrictive policy would invite 21.5% of Dutch cohorts 1941–1962, to yield on average a maximum of 1076 LC deaths prevented per year in the next 2 decades, further extendable with effective smoking cessation policies. About 15–20% of benefits apply to eligible individuals that already had stopped smoking before baseline. Policy makers therefore should imminently consider the implementation of LDCT LC screening in Europe, which may be valuably supplemented but not effectively substituted by primary prevention interventions.

## Contributors

All authors were responsible for the conceptualization of the analysis, interpretation of the data and the revision of the manuscript. Koen de Nijs and Kevin ten Haaf were responsible for development of the methods and verification of the results. Koen de Nijs was responsible for formal analysis, data curation and writing of the original draft. Carlijn van der Aalst, Harry de Koning and Kevin ten Haaf were responsible for funding acquisition.

## Data sharing statement

Data used as input for the MISCAN-Lung model can be requested from the primary sources, as specified in the methodological supplement. Model outcome data can be made available upon reasonable request.

## Declaration of interests

Koen de Nijs, Kevin ten Haaf and Harry de Koning have received funding from the National Institute of Health. Koen de Nijs and Kevin ten Haaf have received funding from the Swiss Cancer Screening Committee. Kevin ten Haaf has received funding from the Dutch Research Council/Netherlands Organization of Health Research (ZonMW). Carlijn van der Aalst reports speaker fees from KALCIO Healthcare and Longkankernet, and travel support from the WHO. Carlijn van der Aalst is advisory committee member for B3Care and SOLACE. Kevin ten Haaf reports grants from Cancer Research UK, Cancer Australia, MSAC Australia, Convergence (Open Mind call), as well travel fees from the CHUV Lausanne and the Rescue Lung society. Harry de Koning reports speaker fees from Menarini and AstraZeneca. Harry de Koning is chair of a European Council committee on improvement in cancer screening in the EU.
