# Supplementary material for: Projected effectiveness of lung cancer screening and concurrent smoking cessation support in the Netherlands
Source: eClinicalMedicine. 2024 Apr 8;71:102570. doi: 10.1016/j.eclinm.2024.102570 (PMC11133792; doi:10.1016/j.eclinm.2024.102570)
Supplement: Supplementary data [file mmc1.docx]

# Supplement to *Projected effectiveness of lung cancer screening and concurrent smoking cessation support in the Netherlands*

Koen de Nijs^1*^, Kevin ten Haaf^1^, Carlijn van der Aalst^1^, Harry J. de Koning^1^

*Corresponding Author [k.denijs@erasmusmc.nl](mailto:k.denijs@erasmusmc.nl)

1. Department of Public Health, Erasmus MC - University Medical Center Rotterdam, 3015 CE Rotterdam, The Netherlands

Inhoud

[Supplementary Results 1](#_Toc158803196)

[Methodological Supplement 1](#_Toc158803197)

[Model summary and structure 1](#_Toc158803198)

[Smoking history generator 2](#_Toc158803199)

[Death from other causes 3](#_Toc158803200)

[Smoking dose-response relationship for lung carcinogenesis 3](#_Toc158803201)

[Natural History of Lung Cancer 3](#_Toc158803202)

[Computed Tomography Screening 4](#_Toc158803203)

[Smoking Cessation Interventions 5](#_Toc158803204)

[Integrating Modules 6](#_Toc158803205)

[Model Outcomes 6](#_Toc158803206)

[Dutch smoking behavior and lung cancer epidemiology 9](#_Toc158803207)

[NELSON Screening Effectiveness 11](#_Toc158803208)

[References 12](#_Toc158803209)

# Supplementary Results

**Supplementary Table 1 -** **Benefits and harms of lung cancer screening per the 4ITLR-THE-LUNG-RUN^1^ strategy, assuming 50% attendance.** *Smoking status as determined at the simulated start of screening (2022), including never-eligible smokers. § Ever-eligible for screening at the start of screening (2022), including former smokers. † Biennial screening ages 60-79 for those with at least 35 packyears or 2.6% PLCOm2012 risk, and maximally 10 years of smoking cessation. ‡ Lung Cancer. ¶ Low-dose Computed Tomography. #:For the population-wide results, we also present outcomes for the scenario with pharmacotherapy smoking cessation support, applied at every screening round. For this scenario, overdiagnosis is measured as excess incidence relative to the scenario with only smoking cessation support (no screening). Results are generated for cohorts 1942-1961, with cohort sizes matched to 2021 populations by birth-year and sex for the Netherlands. Together these cohorts represent 3.85 million people as of 2022. Outcomes are tallied for the entire simulated lifetimes from 2022 onwards. The 50% attendance rate is applied equally across screening (and integrated screening cessation intervention) events.

|  | All Those Eligible for Screening§ | | All Current Smokers* | | All Former Smokers* | | Population-Wide | | |
| --- | --- | --- | --- | --- | --- | --- | --- | --- | --- |
|  | **No Screening** | **4ITLR^†^** | **No Screening** | **4ITLR^†^** | **No Screening** | **4ITLR^†^** | **No Screening** | **4ITLR^†^** | **4ITLR^†^ + Cessation**^#^ |
| LC^‡^ Cases | 146,275 | 153,291 | 110,475 | 114,133 | 111,706 | 112,668 | 239,899 | 244,519 | 243,272 |
| Stage I-II | 23% | 33% | 23% | 34% | 24% | 27% | 23% | 30% | 34% |
| Stage III-IV | 77% | 67% | 77% | 66% | 76% | 73% | 77% | 70% | 66% |
| LC^‡^ Deaths  (reduction) | 139,080 | 125,676  (-9.6) | 104,988 | 94,150  (-10.3%) | 98,746 | 96,180  (-2.6%) | 218,408 | 205,004  (-6.1%) | 204,162  (-6.9%) |
| LC^‡^ Deaths Averted |  | 13,404 |  | 10,838 |  | 2,566 |  | 13,404 | 14,246 |
| Overdiagnosed Cancers  (% of LC cases) |  | 4,620 (3.1%) |  | 3,658 (3.2%) |  | 962 (0.9%) |  | 4,620 (1.9%) | 3,373 (1.3%) |
| False Positives |  | 20,437 |  | 12,827 |  | 7,610 |  | 20,437 | 20,468 |
| Life-years  (increase) | 13.8m | +136,300 (1.0%) | 9.65m | +110,551 (1.1%) | 42.0m | +25,749 (0.1%) | 72.6m | +136,300 (0.2%) | +162,807  (0.2%) |
| Life-years gained per death averted | - | 10.17 | - | 10.2 |  | 10.0 | - | 10.2 | 11.4 |
| CT^¶^ Screens |  | 1.81m |  | 1.16m |  | 0.65m | - | 1.81m | 1.81m |
| Screens per life-year gained |  | 13.3 |  | 10.5 |  | 25.3 | - | 13.3 | 11.1 |
| Proportion Eligible |  | 100% |  | 74.2% |  | 17.5% | - | 21.5% | 21.5% |

# Methodological Supplement

## Model summary and structure

To estimate the effectiveness of lung cancer screening with and without smoking cessation support, we used the MIcrosimulation SCreening ANalysis (MISCAN) Lung model, a stochastic, microsimulation model. In brief, the model simulates individual life histories in the considered population from birth until death, in the presence or absence of a screening program. Through comparing the life histories in the presence of screening with the corresponding life histories in the absence of screening, MISCAN-Lung can estimate the effectiveness and costs of screening scenarios. MISCAN-Lung was calibrated to individual-level data from the National Lung Screening Trial (NLST) and the Prostate, Lung, Colorectal, and Ovarian Cancer Screening Trial (PLCO),^2^ ^3^ and has since been recalibrated to the Nederlands-Leuven Longkanker Screening Onderzoek (NELSON).^4^ Estimates of the preclinical sojourn time by stage, and the probability of lung cancer detection are now matched to outcomes from the NELSON trial, per methods elaborated on in a future section. The model is programmed in Python 3.

MISCAN-Lung is a semi-Markov model, which generates durations for each state. Individuals are simulated one at a time, which allows future state transitions to depend on past transitions giving the model a “memory”. MISCAN-Lung simulates sequences of events by drawing from distributions of probabilities/durations, which makes the results of the model subject to random variation. The model is calibrated such that the aggregate of all these individual outcomes matches the smoking behavior patterns and lung cancer incidence across different sexes and birth cohorts of the Netherlands, and the outcomes of the PLCO, NLST and NELSON trials. For smoking behavior prevalences, we use the Dutch Health Survey for years 1989 to 2020 to establish proportions of current, former and never smokers over time for each 5-year birth cohort and sex separately.^5^ Lung cancer incidence is taken from the Netherlands Cancer Registry, covering all incident lung cancers 2000-2020 in the Netherlands by stage and histology of cancer.^6^

MISCAN-Lung consists of several modules: a demography/smoking history generator module, a smoking-dose response module for lung carcinogenesis, a natural history module and a screening module. The workings of each of these modules will be discussed in detail in the coming sections. Afterwards, we discuss how they were adjusted to Dutch cohorts 1942-1961.

## Smoking history generator

First, birth-tables, representative for the population under consideration, are used to draw a date of birth for each simulated individual. Age, sex and five-year birth-cohort specific smoking initiation probabilities, representative for the population under consideration, are used to determine whether an individual initiates smoking and the age of smoking initiation. Upon smoking initiation, persons enter one of five increasing smoking intensity categories. Age, sex, five-year birth-cohort and smoking intensity category specific by averaged number of cigarettes smoked per day are generated for each individual that initiates smoking. If an individual initiates smoking, age, sex and cohort specific smoking cessation probabilities are used to determine whether an individual ceases smoking and the age of smoking cessation. The workings of additional smoking cessation interventions, which may cause smoking cessation in excess of the background rate, are discussed in the section `smoking cessation interventions`.

## Death from other causes

Upon generating a person’s smoking history, the age of death from causes other than lung cancer is generated, using mortality probabilities based on the person’s smoking history (smoking duration, smoking intensity category and average number of cigarettes per day, smoking status and years since cessation, if applicable), year of birth, age and sex. For example, for the Male 1960 cohort, Figure 1 shows the mortality rate for never-smokers relative to current smokers for each smoking intensity quintile. After smoking cessation, individuals are assigned a mortality probability in between the never- and current-smoekr mortality probability, weighted by their years since smoking cessation. The maximum age an individual can achieve in MISCAN-Lung is exactly 100 years.

Figure 1 - Other-Cause Mortality Probability by Age and Smoking Status for the Male 1960 cohort

## Smoking dose-response relationship for lung carcinogenesis

The smoking-dose response module allows modeling lung carcinogenesis as a function of a person’s age, gender and smoking history. MISCAN-lung utilizes the two-stage clonal expansion model (TSCE) as described by Heidenreich et al., as its smoking-dose response module (which estimates a person's risk of lung cancer, as a function of age and smoking history).^7^ The parameters of the TSCE were obtained through calibration to the Nurses’ Health Study and the Health Professionals Follow-up Study ^8^, and were further calibrated to Dutch lung cancer incidence, smoking behavior, and individual-level outcomes from the NELSON study. ^5, 6^

## Natural History of Lung Cancer

Lung cancers are assumed to progress sequentially through stages IA to IV, as shown in Figure 2. The probability that a lung cancer progresses to a more advanced preclinical stage or is diagnosed clinically (e.g., diagnosed due to symptoms) is modelled by histology and stage. After clinical diagnosis, lung cancer survival is simulated using sex-, stage-, and histology specific survival estimates, obtained from the Netherlands Cancer Registry.^6^ The date of death for individuals with lung cancer is set to the earliest simulated date of death (either due to lung cancer or other causes).


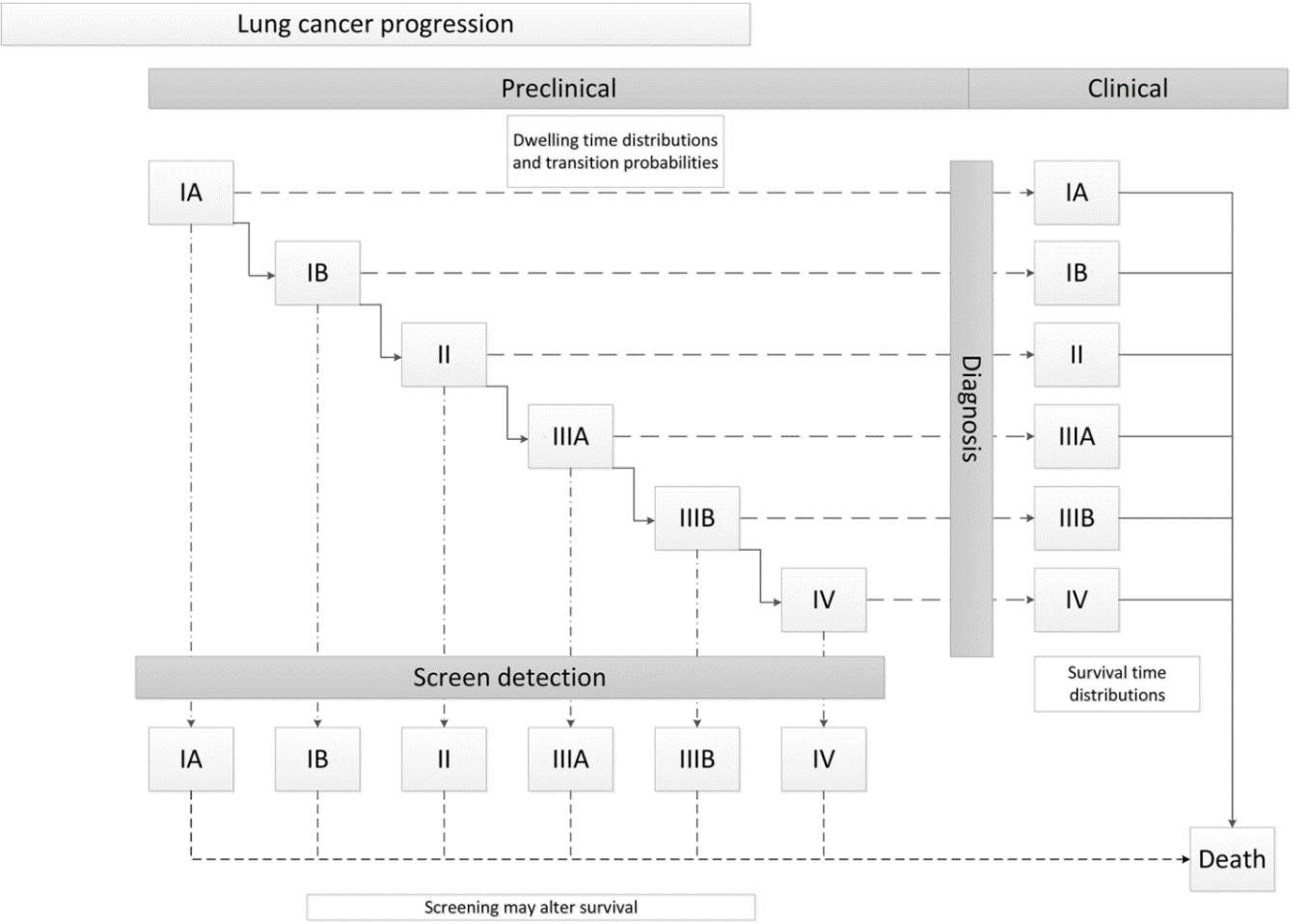


Figure 2 Lung cancer progression in the MISCAN-Lung model

Figure notes: Once lung cancer has developed, it will progress from less advanced to more advanced preclinical stages until it is clinically detected. This process is similar for all histologies, however, the average time spent in the current state differs by histology, preclinical cancer stage and gender. The probability that a cancer progresses to a more advanced preclinical stage or is diagnosed clinically (e.g., diagnosed due to symptoms) is modelled by histology and stage. Screening may detect cancers in each of the preclinical screen-detectable states, depending on the sensitivity of the screening test for the specific histology and preclinical detectable state. Upon detection of lung cancer by screening, a person’s life history may be altered. Detection by screening may prevent the lung cancer death, allowing them to resume their normal (lung cancer free) life history. The probability of lung cancer mortality prevention differs by the stage of detection. After clinical detection the patient’s duration of survival follows a histology and stage specific survival function, which is piecewise uniformly distributed. A screen detected case without successful mortality prevention is assigned the same age of lung cancer death from the life history without screening (in which the cancer is detected clinically at a later age). A person may also die from causes other than lung cancer.

## Computed Tomography Screening

Screening may detect cancers in each of the preclinical screen-detectable states, depending on the sensitivity of the screening test for the specific histology and preclinical stage. The model parameters for CT sensitivity by preclinical stage and histology and the effectiveness of CT screening were calibrated to individual-level data from the NELSON trial.^4^ Upon detection of lung cancer by screening, a person’s life history may be altered. Detection by screening may prevent the lung cancer death, allowing them to resume his normal (lung cancer free) life history. The probability of successful mortality prevention differs by the stage at detection, and was also calibrated to individual-level outcomes from the NELSON study. Negative effects of screening, such as overdiagnosis of lung cancer (described subsequently), are also modelled.

## Integrating Modules

Figure 3 shows an example of how the model integrates the different modules to determine the benefits of screening. The demography/smoking history generator module first generates a date of birth, smoking history and date of death from causes other than lung cancer. This creates a life-history in the absence of lung cancer for Person 1 (shown in life history 1). The smoking-dose response module uses the simulated smoking history to determine whether and when lung carcinogenesis occurs for Person 1 (shown in life history 2). After lung carcinogenesis occurs, the natural history model generates the progression of the cancer, which is diagnosed because of symptoms in stage II in this example and results in a death due to lung cancer, before the death due to causes other than lung cancer would have occurred (shown in life history 1). In the screening module, a screening examination is simulated, as indicated by the arrow (shown in life history 3). The cancer is detected at the examination and, in this case, the earlier detection allows for successful treatment of the cancer. As a result, the lung cancer death is prevented, and the person’s life is prolonged.

Screening may also cause harms, as shown for Patient 2 in Figure 4. In Patient 2 lung cancer also develops, but the cancer would not have been clinically detected without screening (shown in life history 2). However, the cancer is screen-detected in stage IA during the screening examination simulated in the screening module (shown in life history 3). Thus, in this patient, screening detects a lung cancer that would have never become apparent during the patients’ lifetime if screening had not occurred, resulting in an overdiagnosed case. Thus, for Patient 2 screening does not provide any benefits, but results in life-years with lung cancer care that would not have occurred otherwise (overtreatment).


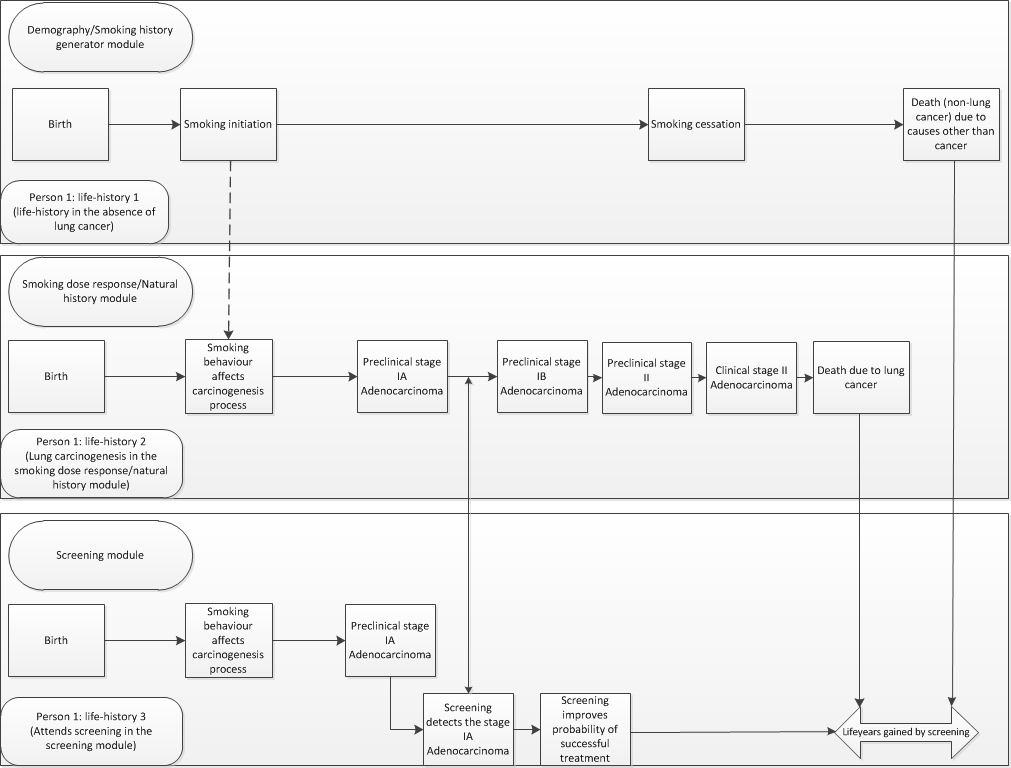


Figure 3 Integrating modules: modelling benefits of screening


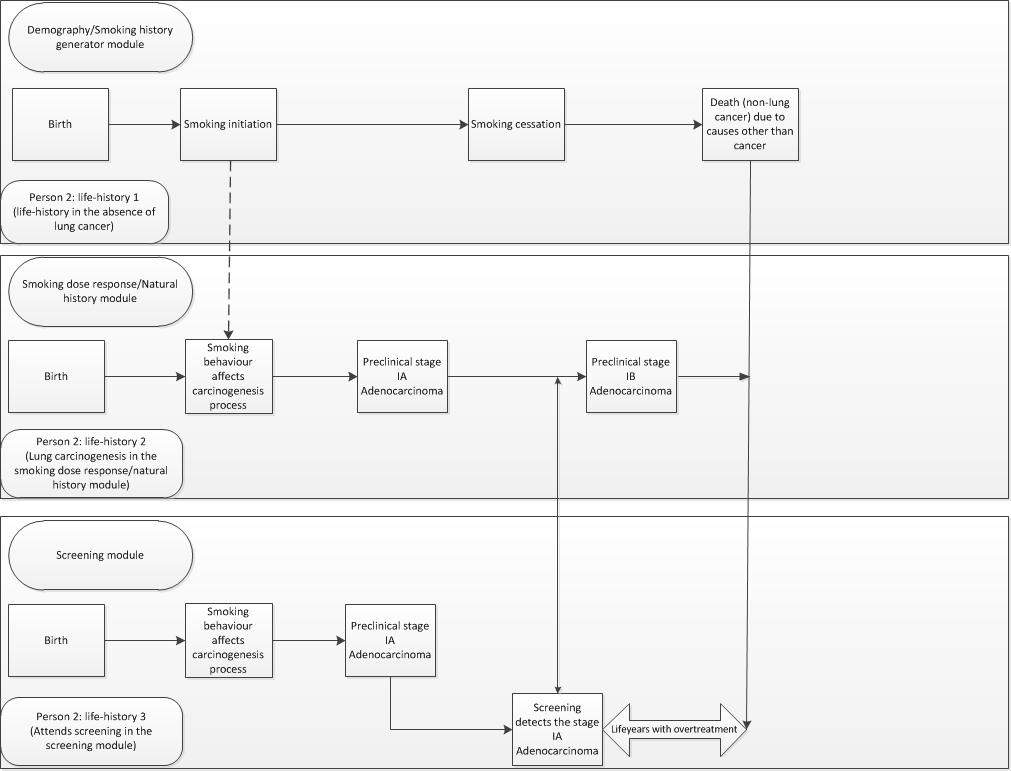


Figure 4 Integrating modules: modelling harms of screening

## Smoking Cessation Interventions

In this particular study, we add a model component to facilitate interventions aimed at inducing smoking cessation. An individual may be scheduled to attend a smoking cessation intervention at a given age. At a probability adjusted for the assumed odds-ratio of the intervention, and the background rate of smoking cessation, the individual may permanently quit smoking instantaneously. The number of cigarettes smoked per day for the years from that point forward is set to 0. The other-cause mortality age and age of lung cancer onset (if no preclinical lung cancer is already present at the age of smoking cessation) originally estimated for the individual for a life-history without smoking cessation interventions are re-estimated to incorporate the change in smoking behaviour. The initial random thresholds of cancer onset and other-cause mortality are preserved, such that the new probability of age can only result in a later age of onset or other-cause mortality.

To obtain the instantaneous probability of cessation from the odds ratio, we multiply the background odds of smoking cessation by the odds ratio, convert these odds to the equivalent overall cessation probability, from which we subtract the background cessation rate. For example, if we have a background cessation rate of 5% and an odds ratio of 1.5 for a given intervention, the background cessation odds are 1 to 19. In the intervention group, we expect odds of 1.5 to 19, or 1 to 12.67, equivalent to a total cessation rate of 7.3%. To get to a cessation rate of 7.3% in the intervention group, we apply an instantaneous cessation probability of 2.4% (1 – 0.927/0.950) at the moment of the intervention.

We obtain odds-ratios by smoking cessation support modality from the literature^9^. We use the odds ratios obtained from a meta-analysis for all studies reporting 6-month point prevalence of smoking status. We forego the 12-month estimates, because although a longer follow-up is associated with more definitive smoking cessation, only a subset of studies report smoking cessation at 12 months. To account for the uncertainty surrounding the smoking cessation odds ratios, we evaluate lung cancer outcomes using the point estimate odds ratios, as well as at the edges of the 95% confidence intervals (CIs) of the reported odds ratios. We include estimates for web-based smoking cessation support (OR 1.14, CI 1.03-1.25), telephone counselling (OR 1.21, CI 0.98-1.50), in-person counselling (OR 1.46, CI 1.25-1.70) and pharmacotherapy (OR 1.53, CI 1.33-1.77).

## Model Outcomes

The model outcomes presented in Table 1 of the main manuscript are generated as follows:

- **LC Cases:** The sum of lung cancer incidence events, including screen-detected lung cancers.
- **LC Deaths:** The sum of lung cancer death events.
- **LC Deaths Averted:** The sum of lung cancer death events, less the sum of lung cancer deaths events in the scenario without interventions (screening or smoking cessation support).
- **Overdiagnosed Cancers:** The number of screen-detected lung cancers that would not have been clinically detected in the scenario without screening. This is calculated by the excess incidence, that is the increase in incidence in the screening scenario relative to the scenario without screening. When smoking cessation interventions are included, it is calculated as the excess incidence relative to the scenario with only the smoking cessation intervention and no screening.
- **False positives:** The number of false-positive screening results, per rates adapted from the NELSON trial results, 1.44% of all first screens, 1.06% of all subsequent screening rounds.
- **Life-years:** Remaining total life-years lived measured from the start of screening (2022).
- **Life-years gained per death averted:** Life-years divided by the `LC Deaths Averted`.
- **CT Screens:** Total required Computed Tomography screens.
- **Screens per life-year gained:** `CT screens` divided by `Life-years`.
- **Proportion eligible:** The share of the given population who are eligible for at least one round of CT screening from 2022 onwards.

## Dutch smoking behavior and lung cancer epidemiology

To calibrate the MISCAN-Lung model inputs to the Dutch setting, we evaluated the model’s potential to replicate Dutch lung cancer incidence. This ensures that model is representative of broader lung cancer epidemiology in the Netherlands.

We simulate lung cancer outcomes for the years 2000-2020 for cohorts 1935 to 1979. Smoking initiation, cessation and smoking-related other-cause mortality are calibrated to cohort life tables from the bureau of statistics and smoking prevalence per the Dutch Health Survey (1989-2020).^6, 10^ Microdata from the Dutch Health Survey informs our estimates of the quintiles of smoking intensity in cigarettes per day, by sex and 5-year cohort.^5^ National tobacco sales contemporary to the Dutch Health Survey are used to evaluate quantity underreporting by health survey respondents, yielding an estimate of 21% underreporting, consistent with previous estimates for other contexts^11-13^. Together, these estimates of smoking behavior are used to inform the Smoking History Generator component of the MISCAN-Lung model. The fit of the smoking history generator to observed current smoking prevalence over time by cohort are given in Figures 5 and 6.

For a given set of model inputs, 10,000,000 life histories are simulated. The sizes of the individual cohorts constituting this population are set to comply to 2010 cohort sizes per Statistics Netherlands. For each simulated individual, a smoking history and smoking related other-cause date of death are drawn. Depending on the smoking history, a lung cancer natural history may be established, using the Two-Stage Clonal Expansion model. Lung cancer outcomes are noted for the years 2000-2020 to compare to population-level calibration targets of lung cancer incidence. Simulated and recorded outcomes include lung cancer incidence and mortality by histology (adenocarcinoma, squamous cell carcinoma, other non-small cell lung cancer, and small cell lung cancer) and stage of cancer at incidence (stages IA, IB, II, IIIA, IIIB and IV). Figure 7 shows the lung cancer incidence generated after model calibration by the MISCAN-Lung model for the period 2000-2020, relative to observed values from the Dutch Cancer Registry.

**Figure 5 - MISCAN-Smoking History Generator (SHG) predictions of current smoking prevalence compared to observed Dutch Health Survey smoking prevalence for Male 10-year cohorts 1930-1970**


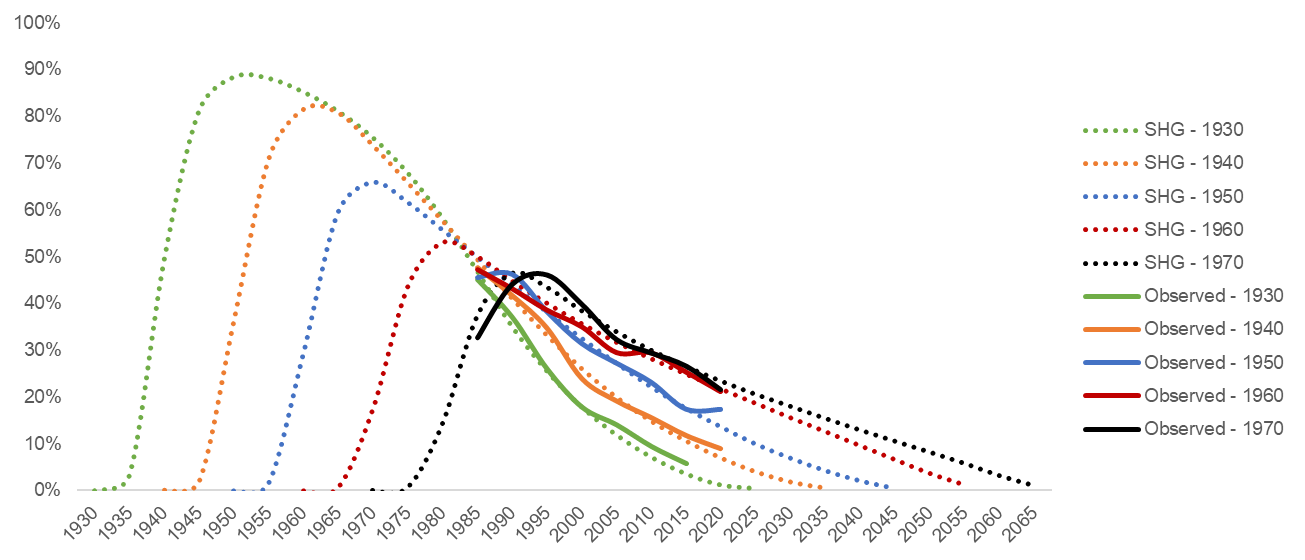


**Figure 6 - MISCAN-Smoking History Generator (SHG) predictions of current smoking prevalence compared to observed Dutch Health Survey smoking prevalence for Female 10-year cohorts 1930-1970**


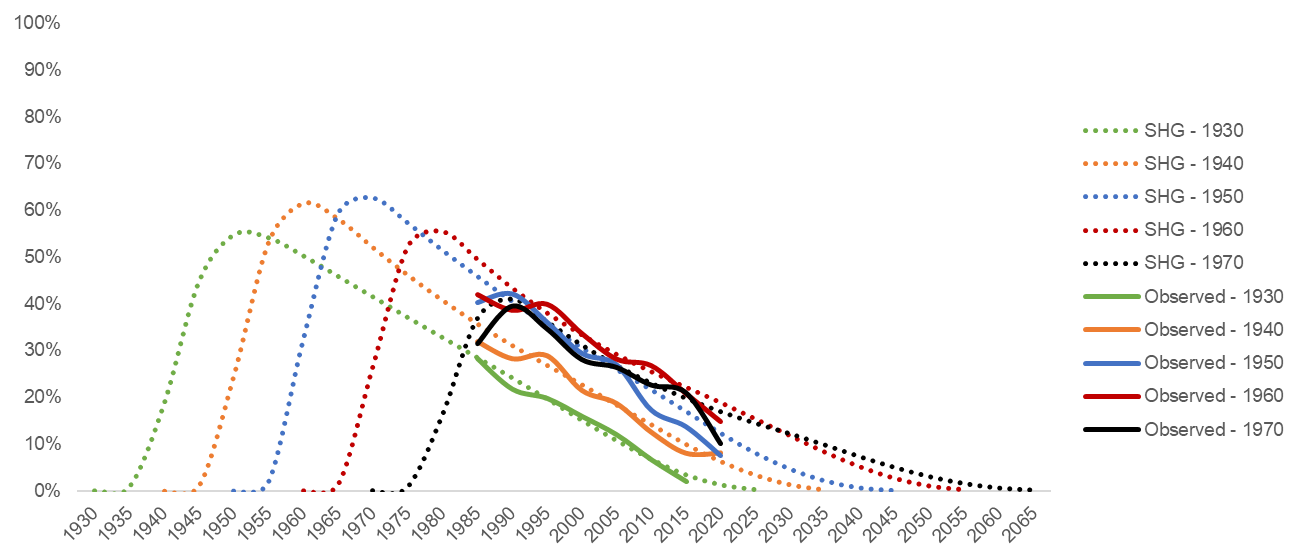


**Figure 7 - MISCAN-Lung simulated lung cancers by age and sex for the period 2000-2020 in the Netherlands.**


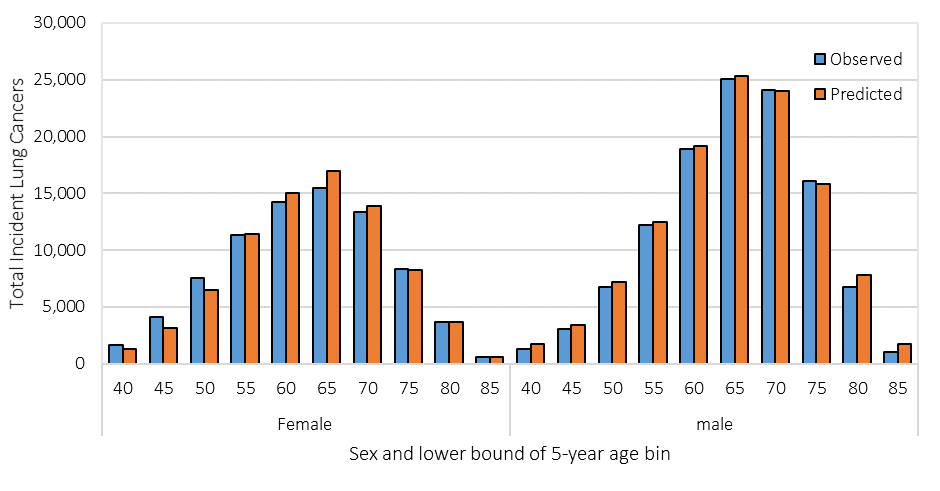


Figure 7 reports MISCAN-Lung predicted gross lung cancer incidence for the period 2000-2020 for the country of the Netherlands, summed across males and females. Dutch health survey data on smoking behaviour by sex for the 1930 to 1979 cohorts is used to inform the MISCAN-Lung microsimulation of life histories. Cohort sizes are based on the 2020 population composition of the Netherlands.

## NELSON Screening Effectiveness

Additionally, the model was calibrated to individual-level outcomes from the NELSON study to re-estimate parameters of screening effectiveness first calibrated to results from the NLST. ^2, 4^ The model calibration replicates the life history of each NELSON participant, and sets the MISCAN-Lung parameters of screening effectiveness to the values that best match the observed outcomes in the NELSON trial. Self-reported smoking histories were used to replicate lung cancer outcomes among simulated NELSON participant. Recalibrated parameters include the sensitivity of the CT screen by stage, as well as the preclinical sojourn time in stage IA Adenocarcinoma. NELSON calibration results suggest a higher sensitivity of CT screening for the detection of lung cancer than previous results from NSLT and PLCO trials, as well as a longer period of detectability in stage IA Adenocarcinoma. ^2, 3^ The complete NELSON-adjusted MISCAN-Lung parameter set and calibration methodology are being prepared for presentation in a forthcoming publication.^4^

## References

1. 4-IN THE LUNG RUN: towards INdividually tailored INvitations, screening INtervals, and INtegrated co-morbidity reducing strategies in lung cancer screening, 2020.

2. ten Haaf K, van Rosmalen J, de Koning HJ. Lung Cancer Detectability by Test, Histology, Stage, and Gender: Estimates from the NLST and the PLCO Trials. *Cancer Epidemiology Biomarkers &amp;amp; Prevention* 2015;**24**: 154.

3. Meza R, ten Haaf K, Kong CY, Erdogan A, Black WC, Tammemagi MC, Choi SE, Jeon J, Han SS, Munshi V, van Rosmalen J, Pinsky P, et al. Comparative analysis of 5 lung cancer natural history and screening models that reproduce outcomes of the NLST and PLCO trials. *Cancer* 2014;**120**: 1713-24.

4. de Nijs K, ten Haaf K, van der Aalst CM, Oudkerk M, de Koning HJ. OA05.04 A Comparison of Stage- and Histology-Specific CT Sensitivity in the NELSON Trial and the NLST. *Journal of Thoracic Oncology* 2022;**17**: S14.

5. Statistics Netherlands. Dutch Health Survey, 1989-2020.

6. Netherlands Cancer Registry (NCR). Lung Cancer Incidence and Survival. In: Netherlands Comprehensive Cancer Organisation (IKNL), ed., 2000-2012.

7. Heidenreich WF, Luebeck EG, Moolgavkar SH. Some properties of the hazard function of the two-mutation clonal expansion model. *Risk Anal* 1997;**17**: 391-9.

8. Meza R, Hazelton WD, Colditz GA, Moolgavkar SH. Analysis of lung cancer incidence in the nurses’ health and the health professionals’ follow-up studies using a multistage carcinogenesis model. *Cancer Causes & Control* 2008;**19**: 317-28.

9. Cadham CJ, Jayasekera JC, Advani SM, Fallon SJ, Stephens JL, Braithwaite D, Jeon J, Cao P, Levy DT, Meza R, Taylor KL, Mandelblatt JS, et al. Smoking cessation interventions for potential use in the lung cancer screening setting: A systematic review and meta-analysis. *Lung Cancer* 2019;**135**: 205-16.

10. Statistics Netherlands. Levensverwachting; geslacht, leeftijd (per jaar en periode van vijf jaren), 2020.

11. Pérez-Stable EJ, Marín BV, Marín G, Brody DJ, Benowitz NL. Apparent underreporting of cigarette consumption among Mexican American smokers. *American Journal of Public Health* 1990;**80**: 1057-61.

12. Gallus S, Tramacere I, Boffetta P, Fernandez E, Rossi S, Zuccaro P, Colombo P, La Vecchia C. Temporal changes of under-reporting of cigarette consumption in population-based studies. *Tobacco Control* 2011;**20**: 34-9.

13. Liber AC, Warner KE. Has Underreporting of Cigarette Consumption Changed Over Time? Estimates Derived From US National Health Surveillance Systems Between 1965 and 2015. *Am J Epidemiol* 2018;**187**: 113-9.
